# Closing the collaborative gap: Aligning social and ecological connectivity for better management of interconnected wetlands

**DOI:** 10.1007/s13280-014-0605-9

**Published:** 2015-01-09

**Authors:** Stuart Kininmonth, Arvid Bergsten, Örjan Bodin

**Affiliations:** Stockholm Resilience Centre, Stockholm University, Kräftriket 2B, 106 91 Stockholm, Sweden

**Keywords:** Governance, Socio-ecological system, Wetlands, Network theory, Motif

## Abstract

Understanding how governance structures align to ecological processes in a landscape is critical for effective management of ecological resources. Ecological resources are not independent from each other, instead they are interconnected, and their well-being is often critically dependent on upholding ecological connectivity, especially in times of change and disturbances. Coordination and collaboration among managing actors, each managing their own piece of the puzzle, is therefore essentially a requirement for effective management. We present a conceptual model that includes ecological resources, managing and coordinating actors, along with an explicit representation on how all these entities are connected to each other. We apply this model to 25 municipalities that manage 408 wetlands in central Sweden. The study shows a good social and ecological alignment, however with a high prevalence for coordination through third parties. We discuss this pattern emergence, its potential implications, and examine which municipalities adopt these coordinating functions.

**Electronic supplementary material:**

The online version of this article (doi:10.1007/s13280-014-0605-9) contains supplementary material, which is available to authorized users.

## Introduction

Understanding how governance structures affect the resilience of social–ecological systems is critical for conservation (Garmestani and Benson 2013) especially under the looming threat of climate change (Folke et al. 2005; Folke 2006; Galaz et al. 2008). The Millennium Ecosystem Assessment (2005) confirms that over the past 50 years, humans have changed ecosystems more rapidly and extensively than in any comparable period of time in human history. The challenge of reversing this rapid degradation of ecosystems while meeting increasing demands for ecosystem services will involve significant changes in policies, institutions, and practices (Millennium Ecosystem Assessment 2005). A key contributor to better ecosystem management is to develop effective governance structures that are aligned to both social and ecological factors of importance (Young 2002; Folke et al. 2007; Biggs et al. 2012), yet more explicit evaluation of what constitutes effective governance structures is required (Cumming et al. 2006; Guerrero et al. 2013, 2014).

Governance structures that facilitate the development of knowledge and adaptive management practices, support multilevel governance systems that align with the scales of the managed ecosystems, and that can deal with uncertainty and surprise are needed to ensure effective and sustainable governance of coupled social and ecological systems (Young 2002; Folke et al. 2005; Galaz et al. 2008; Ekstrom and Young 2009). Governance structures in this context relate to the actors that have a direct and/or indirect effect on the ecological system and the institutions and inter-actor relationships that affect the behaviors of these actors. Overarching principles for effective governance have been suggested (Biggs et al. 2012) but often lack effective operationalization. Aligning the governance structure to reflect the underlying ecological processes requires additional attention (Pelosi et al. 2010). Cumming et al. (2006) outline the need for flexible institution arrangements that can reorganize in response to ecological processes. This includes measures to broadening the scope (e.g., geographically) of the governing arrangements to accommodate large-scale ecological processes. Options for natural resource managers to align governing arrangements with larger ecological scales include enlarging participation either directly with associated managers or through a coordinating partner.

Studies scrutinizing the effectiveness of governance structures, containing the complicated interplay of governing actors and ecological resources, are required. Since the level of complexity of coupled social–ecological systems is often very high, explicit empirical investigations on exactly what governance structures are effective or not in governing specific ecosystems are inherently difficult. However, within a network analytical perspective, the identification of some archetypical governance substructures that are beneficial (or detrimental) for effective governance of social–ecological systems would be helpful in further understanding what constitutes effective governance (cf. Senge 1990). In Bodin and Tengö (2012), a set of basic governance substructures in a rural agricultural setting were described as highly simplified social–ecological subsystems (i.e., networks) only consisting of two actors and two ecological resources (motifs), and certain motifs were shown, using relevant social and ecological theories, to seemingly be effective in preserving well-functioning ecosystems. Conceptually similar, McAllister et al. (2013) highlighted that, based on specific motif distributions, actors (local government) tended to be collaborative within closed group but adopted an advocacy role when situated in less cohesive groups. This result was supported by qualitative interview-based analysis. Both these studies demonstrate, using two very different study systems, the applicability of studying a complex governance system as being composed of sets of simplified network substructures (motifs).

While the social–ecological system and the representative social–ecological network are often complex in their entirety, the statistical distribution of some specific substructures (motifs) can be utilized as a mechanism to understand the structural character of the complete system (Robins et al. 2007). Comparing the prevalence of some theoretically motivated motifs across the entire system to their occurrence in randomized versions of the same system enables the researcher to comment on the functional character of the whole system (Handcock 2003). In particular, frequency deviations of a particular motif in an empirical network compared to a large set of randomized study systems can provide support for structural interpretation (Bodin and Tengö 2012).

To illustrate the applicability of this approach, we investigate the governance structure of wetland management in a peri-urban landscape in Sweden where we model the municipalities as actors (social nodes) and aggregations of wetlands as ecological resources (ecological nodes), all being intertwined in a complex social–ecological network.

Wetlands require appropriate management to ensure they remain resilient especially given the external stress of land use alterations and climate change (Graymore and McBride 2013). Their ecological resilience is partially derived from the interconnected processes of colonization by different species, meaning that species persistence in any of the wetland patches, and ultimately in the whole landscape, is reliant on the possibility of species to disperse between the wetlands. This dispersal satisfies evolutionary, metapopulation, and range expansion impetuses and highlights the ecological need to conserve these fragile habitats (Tulbure and Broich 2013; McIntyre et al. 2014). Amphibians, in particular, are useful in highlighting the dependency between wetland health and ecosystem services such as disease mitigation (Ounsted and Madgwick 2008). While the ecosystem services delivered by wetlands are diverse and internationally recognized (Mooney et al. 2005; Costanza et al. 2006), wetland systems are often severely fragmented as a result of widespread draining to increase agricultural and forest productivity (Zedler and Kercher 2005). Protecting or restoring wetland connectivity often requires interaction among managing agencies, since the demarcation of individual governance boundaries rarely reflects broader scale wetland ecological connectivity (Bergsten 2014). In this perspective, joint management or third-party coordination between actors managing different patches of wetlands represent governance arrangements that are potentially capable of upholding the function and connectivity of wetlands, at multiple levels, from the local patch to regional ecosystems.

In this paper, we investigate a selected suite of motifs that cover a range of ineffective to effective archetypical governance structures influenced by the governance principles of Biggs et al. (2012)(i.e., managing connectivity) and the general principle of social and ecological process alignment (Young 2002; Folke et al. 2007; Galaz et al. 2008) (i.e., matching institutional interactions to environmental processes) and common-pool resource theory (Ostrom 1990) (i.e., natural resource sharing dilemma). These motifs capture the essential elements of governance structures conducive for effective management of shared or interconnected ecological resources. Hence, we focused our study to two overlapping but different governance challenges; when two or more actors have one specific resource in common, or when they each manage different ecological resources that are interconnected. In addition, we outline a conceptual approach for modeling complex governance arrangements especially where ecological connectivity is important. We then apply this model to study the wetland management in central Sweden. Finally we discuss the results with emphasis on the model’s capacity and implications for application in governance of social–ecological systems.

## Materials and methods

In this section, we describe the motif-based analytical framework for analyzing the problem of governance alignment (‘fit’ as defined by e.g. Young 2002 and Folke et al. 2007) in coupled social–ecological systems, followed by the application of the framework to a regional example in Sweden.

### The coordination motif

**Fig. 1:**
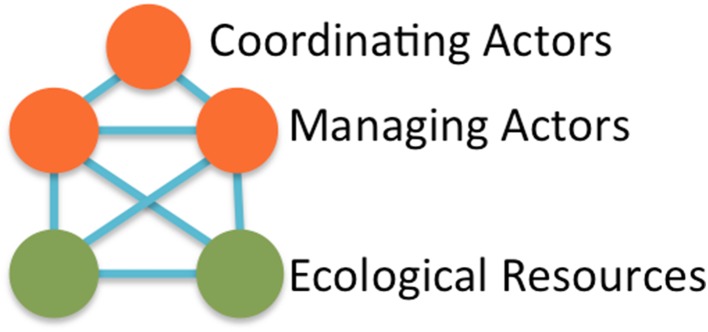
The fully connected five-node governance motif (assuming the coordinating actor is not directly connected to the ecological resources)

**Fig. 2:**
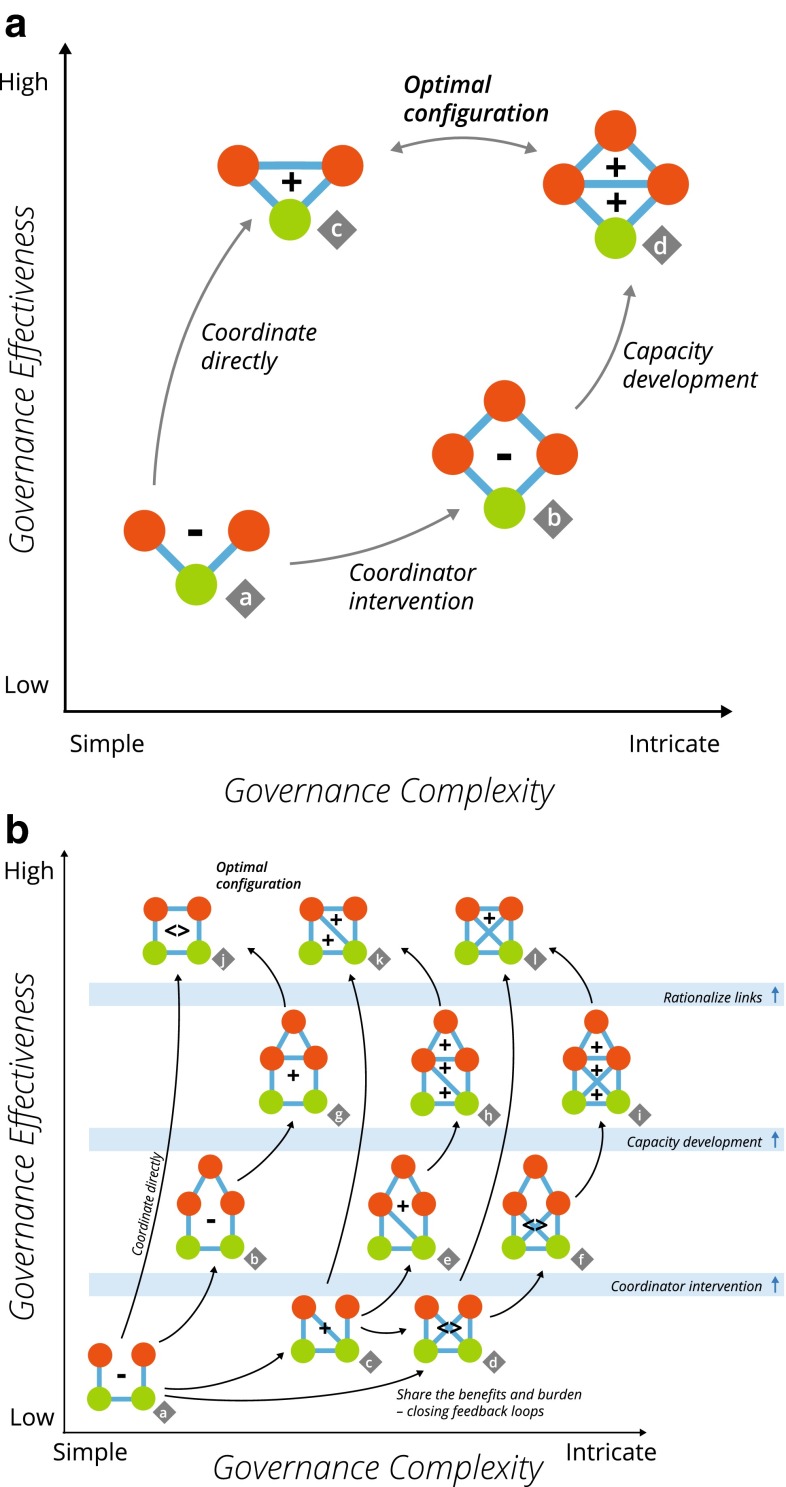
**a** The common resource pool motif subset (*a*–*d*) display across effective complexity space. **b** Connected resource motif subset (*a*–*l*). The observed occurrences measured as the number of standard deviations from the mean of the random simulations are indicated by the +/−/= signs (+ equals 1–5 sd Greater, ++ equals more than 5 sd Greater, − equals 1–5 sd Less, − − equals more than 5 sd Less and < > equals Same; Table 3)

The basic actor–resource interaction model (Bodin and Tengö 2012; Bodin et al. 2014) is described by a simple graph structure that has nodes symbolizing the actors and the ecological resources (Fig. 1). Connecting lines represent the interrelations between the ecological resources, between actors and ecological resources, and between actors. In this regard, our basic motif (Fig. 1) is expanded from Bodin and Tengö (2012) to include five nodes (three social and two ecological). However, it should be noted that our study also includes the subsets whereby, at the most basic level, two actors can manage a single ecological feature (i.e., 3-node motif). Since the full suite of 5-node motif combinations is neither analytically tangible nor desirable for this exercise, we selected a subset comprising only a fraction of all possible motifs (Fig. 2) that describes some social–ecological structures associated with the main governance challenges (accomplishing joint resource management across jurisdiction boundaries) at focus for this study.

**Fig. 3:**
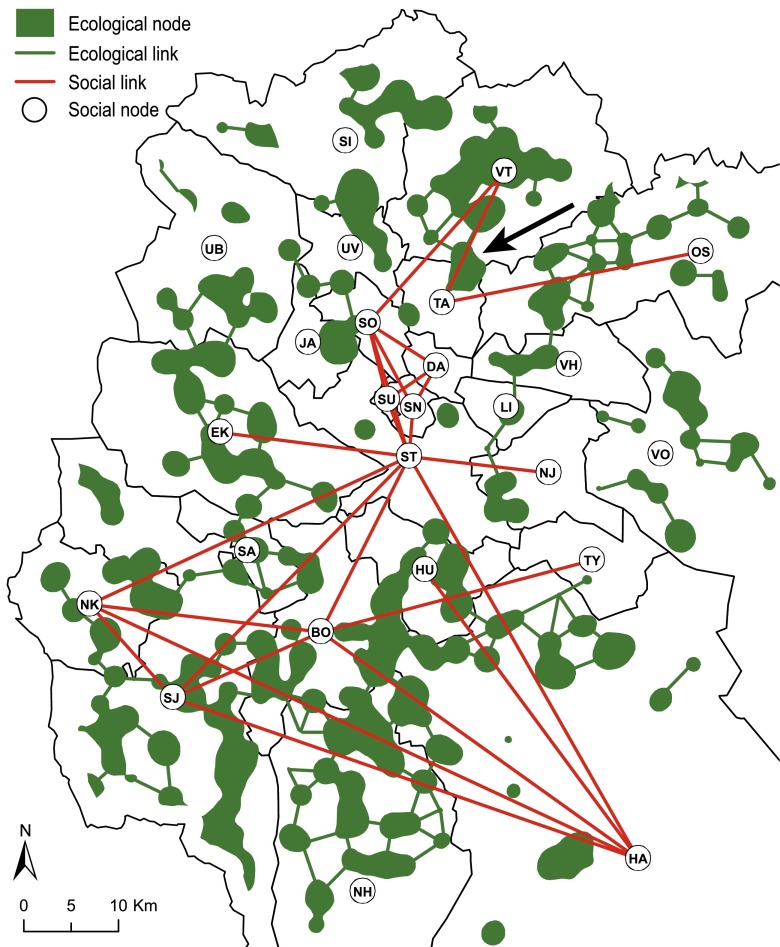
Overview of the Stockholm County wetland case study area with social and ecological links shown. Each ecological node comprises at least 6 ha of wetland. Municipal Council codes are described in Table 1. In addition to the ecological and social links explicitly displayed, there are also socio-ecological links between actors to each ecological node in the municipal jurisdiction. For example, the *arrow* indicates a trans-boundary ecological node, linked to both TA and VT

### Different collaboration and coordination archetypes (motifs)

We developed a conceptual model for the systematic linkage of key motifs for socio-ecological governance (Fig. 2a, b). The model is divided into two parts based on whether the governance challenge stem from the ecological resources being shared (a common-pool resource) or interconnected. Each of the motifs associated with resource sharing or resource connectivity is displayed within a two-dimensional space created by complexity and effectiveness.

We have limited our analysis to motifs containing only two resource managers for the sake of interpretation. The single ecological resource (Fig. 2a) reflects the common pool situation. The simple model contains only four motif types that describe the situations where two resource managers share an ecological resource. The simplest motif is where two social actors jointly manage a resource without indicating any social interaction based on wetland management (motif a), and we assume this to be the least effective configuration according to common-pool resource theory (Ostrom 1990). On the other end of the effectiveness spectrum, we put the motifs where the resource managers are interacting (motif c) as the most effective, while the presence of a coordinating actor (motif b and d) is also assumed to enhance collaboration, albeit in a more complex manner. The primary drivers that link the motifs together are described as ‘coordinator intervention’, ‘capacity development’, ‘rationalize links’, and ‘coordinate directly’. These transitions describe a hypothetical process where an inefficient governance arrangement (motif a) is initially improved through the involvement of a third municipal council that coordinates (connects indirectly) the two managing actors (motif b), who may later engage in joint (direct) communication (motif d). Since communication efforts require resources of time and money, this governance structure could be reduced in complexity through the depreciation of the coordinator links. Conversely, the social actors may decide to coordinate directly as a result of becoming aware of the joint ecological responsibility.

The second model (Fig. 2b) displays 12 motif types (‘a’–‘l’) containing two ecological resources that are interacting and two or three (social) actors. This represents a significant increase in complexity in terms of possible structural combinations, and hence this model only contains a limited set of all possible motifs. The simplest and the most ineffective motif (motif a) describes a situation where there is ecological dependency present (such as dispersal) between two ecological resources yet the governing actors are not coordinating their management or resource use. The model then describes several trajectories that each represents a series of transitions. The intervention of a coordinating actor can foster the direct interaction between actors or act as a broker (motifs b and g). In this second model, we also introduce ‘shared benefits and burdens’, meaning that if one actor is connected to two interconnected ecological resources, that actor will directly experience more systemic “side effects” resulting from mismanagement of either of the resources. For example, assume one manager is connected to two ecological resources that are interconnected. If one of the resources is mismanaged, the resulting degradation could then spread to the other resource through the ecological link. Since the manager is also connected to that resource, the effect of this “dispersed degradation” would also affect the manager. In other words, the manager will, in addition to suffering the direct effect of the mismanagement of the first resource, also experience the indirect effect on the other resource (i.e., the feedback loop is closed). This internalization of dispersed costs and benefits will likely stimulate improved management taking systemic effects into account. However, it also increases complexity and given that potentially not all actors experience the same level of internalization, governance improvements might never materialize.

### Motif analysis

A social–ecological network representing a real system is naturally much more complex than any of the defined building blocks (motifs). However, by extracting all the possible combinations of social actors and ecological resources (social and ecological nodes) from the full-scale social–ecological network, the frequencies of all the different motifs in the real system can be quantified. The outcome of this frequency analysis reveals the relative abundances of the different motifs (Bodin and Tengö 2012). Thus, the frequency analysis gives a precise measure of how commonly any specific motif occurs in the real case. That measure should however be related to a baseline measure, i.e., the measure needs to be grounded. To that end, we related the observed frequency with the frequencies derived in the same way from a large set of randomly generated social–ecological networks. A random social–ecological network (i.e., the null model based on the same number of nodes and social-to-social, social-to-ecological, and ecological-to-ecological links, but without any other distributional limits) thus provided a baseline estimate, and if and to what extent the distributions of building blocks deviate from the baseline informs whether these different building blocks are suppressed or enhanced in the real system.

## Case study: Stockholm County wetlands

The study system comprises 25 municipalities and 408 wetlands in central Stockholm County, Sweden (Fig. 3; Table 1) (Bergsten 2014).

**Table 1:** Municipalities, display codes, wetland number, and total area of wetland (ha)

Municipality	Display code	Wetlands number	Area of wetlands (ha)
Sigtuna	SI	30	960.7
Vallentuna	VT	28	1006.1
Upplands-Bro	UB	17	301.5
Österåker	OS	31	520.1
Upplands Väsby	UV	13	269.1
Värmdö	VO	25	401.4
Täby	TA	6	57.9
Järfälla	JA	2	160
Sollentuna	SO	7	109.9
Vaxholm	VH	6	72.9
Ekerö	EK	19	636.5
Danderyd	DA	1	13.7
Stockholm	ST	11	205
Lidingö	LI	4	28.9
Nacka	NJ	6	112.7
Södertälje	SJ	69	1483
Salem	SA	5	103.3
Huddinge	HU	19	327
Tyresö	TY	6	33.2
Botkyrka	BO	29	824.2
Nykvarn	NK	16	593.7
Haninge	HA	53	1358.3
Nynäshamn	NH	53	1556.6
Solna	SN	0	0
Sundbyberg	SU	0	0

### Social (actor) nodes and links

The social network layer is constituted by municipal councils as nodes (one per municipality) and inter-municipal collaborations as links. We asked the municipalities about their collaborations using a web-based survey in 2011 (100 % response rate). We targeted municipal officials (mainly ecologists and environmental planners) who were involved in wetland management and land use planning, for example by regularly providing ecological advice regarding different land use possibilities. Details regarding what constitute inter-municipal collaborations are described in Electronic Supplementary Material, Section 1.1.

### Ecological (resource) nodes

The ecological network layer is derived from all 408 wetlands in the study area, according to the National Swedish Wetland Survey (Gunnarsson and Löfroth 2009). Wetland species communities are connected at multiple scales, corresponding, for example, to varying dispersal distances of the different species that use wetlands as habitat and to contrasting modes of locomotion for a particular species. For example, juvenile and adult dispersal of an amphibian species may occur at different scales (Richardson 2012). Multi-scale connectivity patterns have been demonstrated for different amphibian species, e.g., for the Western toad *Bufo boreas* (Murphy et al. 2010) and for the European tree frog *Hyla arborea* (Angelone et al. 2011). Our analysis uses amphibians as model species, which is a species group that has previously served as indicators of biological diversity in Stockholm Municipality (e.g., Löfvenhaft et al. 2004). Consequently, amphibian ecology provides a suitable surrogate for socio-ecological assessment of wetland health management at a macro-ecological scale. Although the parameter values likely relate to other taxa than our model species, or to processes other than animal movement, we stress that any applications for management must have the specific species and processes in mind. In contrast, Moor et al. (2014) describe the functional traits of wetland vegetation with direct linkages to ecosystem services at a local scale. Individual municipalities will need to combine both approaches to ensure long-term sustainability of wetland ecosystem services. Further details are described in Electronic Supplementary Material, Section 1.2.

**Fig. 4:**
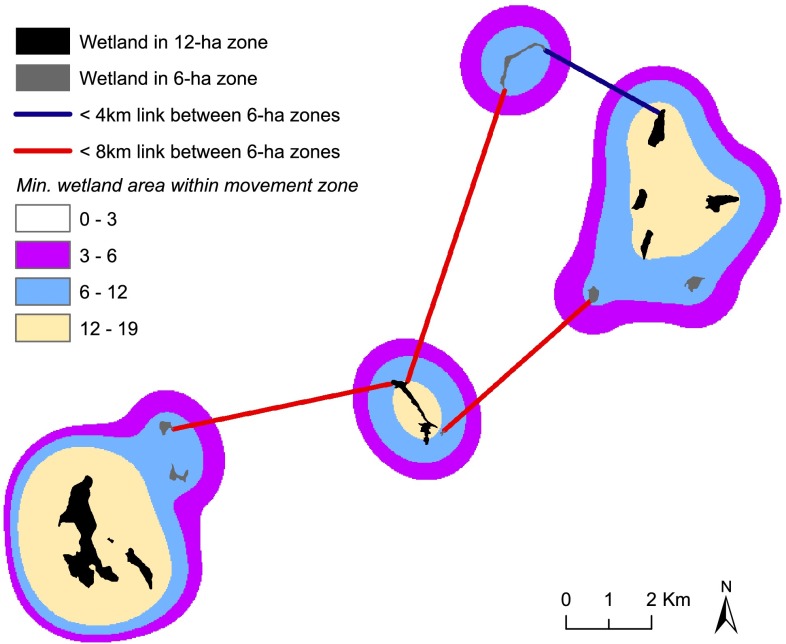
Ecological nodes in a part of the study area. Black wetlands are part of three ecological nodes (movement zones) with >12-ha wetland, whereas nodes with >6-ha wetland include also gray wetlands. For the 4 nodes with >6-ha wetland area, the *blue* link represents connections up to 4 km, whereas *blue* and *red* links connect movement zones up to 8 km. At 13 km, the nodes in the image are fully connected

Based on the wetland area density, we generated three sets of ecological nodes by delineating zones with more than 3-, 6-, or 12-ha wetland (Fig. 4). Ecological links between movement zones were identified when the edge-to-edge distance between wetlands did not exceed 4, 8, or 13 km. Combined with the collaboration data, nine different social–ecological networks were produced (Table 2).

**Table 2:** Estimation parameters for the ecological network layer

Nodes		Links
Regular movement within node	Wetland area within node	Rare dispersal between nodes
Max. 2 km	Min. 3, 6, or 12 ha	Max. 4, 8, and 13 km

### Social–ecological links

Links between the social network and the ecological network represent each municipality’s responsibility to manage each ecological node (movement zone) in its municipal jurisdiction. Movement zones that stretch over several municipalities have multiple social–ecological links, one to each of these municipalities (Fig. 3).

### Wetland analysis with motifs

We used our study system of municipalities and wetlands to explore if and to what extent our motif subset was present. As described earlier, the motif analysis is based on a comparative approach where the empirical network is related to a large set of randomly generated networks. In our analysis, we captured the differences by measuring the deviations in terms of how often the motifs occurred in the empirical network compared to the mean value in the frequency distributions of the random networks (measured in number of standard deviations). We also counted how many times the different municipalities occurred in the different positions in the selected set of motifs (Bodin et al. 2014). These numbers were used in a Principal Component Analysis with the objective to single out different groups or individual municipalities that were similar (or different). Limitations imposed by this methodology, especially in relation to the statistical analysis, are explored in the discussion.

## Results

For each of the nine social–ecological networks, we compared the observed wetland governance structure (25 social nodes, 82–116 wetland movement zones, Electronic Supplementary Material, Fig. S1; Table S1) to 1000 randomly assembled networks and evaluated the frequency of the 16 governance motifs (Electronic Supplementary Material, Fig. S2). We found the general result that motifs representing the lack of appropriate governance coordination were less common than expected for both single and connected ecological resource situations (motif a and b in Fig. 2a, motif a–f in Fig. 2b; approximately 4 standard deviations from the mean of the random distribution; Fig. 2). We also discovered that motifs representing high levels of coordination (2a.c, 2b.j, 2b.k, 2b.l), especially with a coordinator actor (2a.d, 2b.g, 2b.h, 2b.i), were more common than expected (9–100 standard deviations from the mean of the random distribution; Fig. 2) for both single and connected ecological resource situations.

**Fig. 5:**
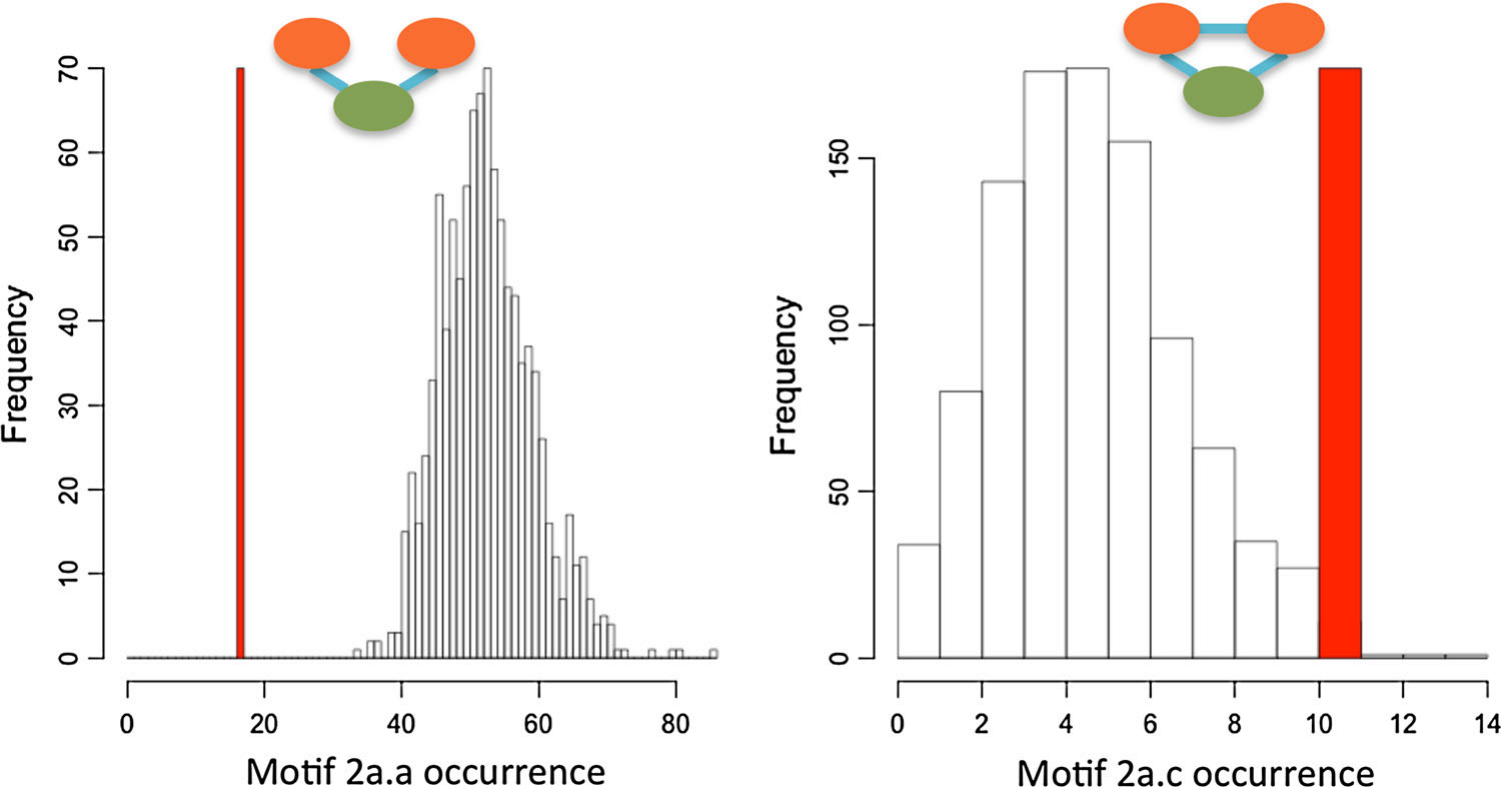
The figure shows the two simplest motifs (2a.a and 2a.c) and their frequency distribution when observed in 1000 random simulations. The *red bar* indicates the single frequency value (bar height only ensures visibility) that was observed for the ecological parameters of 3 ha and 13 km (Table 2). The bar situated to the left of the random distribution indicates that the open motif 2a.a is less frequent that would be expected if randomly assembled. The number of standard deviations (5.3 for 2a.a, 2.7 for 2a.c) reflects the strength of this pattern

The nine combinations of ecological configurations (Table 2) capture a wide range of movement possibilities and habitat area requirements of wetland dependent animals. Yet, our measure of standard deviations of motif frequency from the mean value of the randomly generated networks was consistent (Table 3) across the 9 ecological scenarios (Table 2). Motifs that signify connected governing actors with coordinating actors for both the common-pool resource model (Fig. 2a) and the connected ecological model (Fig. 2b) were observed to occur at a higher frequency than expected (Fig. 5). This illustrates the propensity of the municipal councils to configure themselves toward this governance structure.

**Table 3:** Motif set from Fig. 2 showing range of standard deviation values for the 9 ecosystem scenarios. The count for the number of networks that the motif occurs at least once for 1000 simulations, minimum and maximum values, mean value and range from the number of standard deviations from the random mean to the observed value are shown for each motif indicated by Fig. 2 code

Code	Count	Min.	Max.	Mean
2a.a	1000	−6.7	−5.3	−6
2a.c	996	1	2.8	1.875
2a.b	999	−2.1	−1.3	−1.7
2a.d	451	12.8	16.9	15
2b.a	1000	−9.1	−4.1	−6
2b.c	1000	1.7	8	4.325
2b.d	400	−0.7	7.3	2.175
2b.k	942	15.9	21.2	19.05
2b.l	48	−0.1	9.7	6.625
2b.j	1000	−2.1	2.6	−0.15
2b.b	1000	−3.5	−2.3	−2.9
2b.e	917	1	4.3	2.325
2b.f	78	−0.3	7.2	2.275
2b.g	723	0.6	17.8	6.275
2b.h	204	62	106.9	82.025
2b.i	8	0	79.2	48.45

**Fig. 6:**
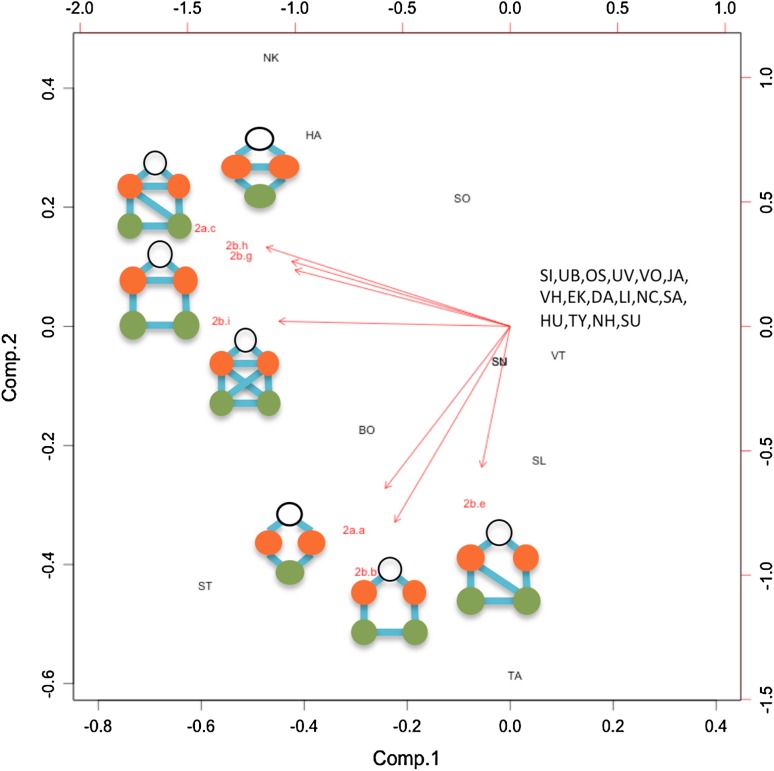
Principal Component Analysis of the municipal councils (Table 1) based on their normalized observed occurrences in occupying a coordinator position across seven motif types (Table 4). *Black circles* indicate the designated coordinator position for each motif. The motif 2b.f was not included as there were no observed occurrences of this type

Examination of the specific position occupied by every social actor for each coordinator motif type provided insights into the functional role occupied (Table 4). For some motif types (e.g., 2b.f), the observed coordinator position was not occupied, while other motifs including a coordinating position (e.g., 2b.g) occurred more frequently (Table 4). Three main coordinator groups were observed and displayed in the principal components analysis (Fig. 6). The motifs characterized by the fully connected social triangle (i.e., the coordinator position links two actors that are already directly connected, such as 2.b.h) describe the municipal councils of Nykvarn and Haninge. Municipal councils that occupy a coordinator position linking two other councils that were *not* directly connected (e.g., 2b.e) characterize Täby council, while Stockholm was a combination of both configurations. The third group of 16 councils was not identified as occupying coordinating positions more or less than at an “average” degree. Two councils Sundbyberg and Solna did not contain wetlands yet were identified as participating as coordinating actors in a small number of cases.

**Table 4:** Observed occurrences of each municipal council for the coordinator positions across eight motifs. Motif codes relate to Fig. 2a, b then the specified letter (‘a’–‘l’). Based on the 3-ha home range and 13-km dispersal threshold model

	2a.b	2a.c	2b.b	2b.e	2b.f	2b.g	2b.h	2b.i
Sigtuna								
Vallentuna			2					
Upplands-Bro								
Österåker								
Upplands Väsby								
Värmdö								
Täby	1		4	5				
Järfälla								
Sollentuna			6					
Vaxholm								
Ekerö								
Danderyd								
Stockholm	2	4	9	1		7	11	2
Lidingö								
Nacka								
Södertälje		3				1	8	
Salem								
Huddinge								
Tyresö								
Botkyrka	1	2	4	1		6	6	1
Nykvarn		5				11	15	1
Haninge		4				7	8	2
Nynäshamn								
Solna		1	4			2	3	
Sundbyberg		1	4			2	3

## Discussion

The conceptual model that links together the motifs describing the shared ecological resource governance is presented and provides a novel approach to the structural analysis of socio-ecological systems. Combining the generic governance principles with specific network building blocks enables detailed interrogation of the position that social actors occupy and the governing structure apparent for each ecological resource. This detailed examination of governance provides an organizational methodology to address issues of social–ecological fit (Young 2002; Folke et al. 2007; Galaz et al. 2008). An important contribution compared to previous work is that we provide the systematic inclusion of a third social actor occupying a coordinating position.

Application of this conceptual model to a wetland governance system in Sweden provides insights to distribution of governance roles and structures across a varied and complex social–ecological landscape. There is a gradient going from the underrepresentation of the least favorable motifs, for both models (Fig. 2), to an overrepresentation of the motifs better structured to favor collaborative governance of shared and/or interconnected ecological resources. This suggests that the municipalities in Stockholm county have both identified the need for, and assigned priority for, local-scale coordination of wetland management for the benefit of the studied model organisms. The 5-node motifs, that describe the potential facilitation role of actors occupying coordinator positions, are more commonly observed than expected randomly. Admittedly this could be a partial effect of a basic social process, i.e., two actors connected to a common third actor tend to, over time, also connect to each other (transitivity; see e.g., Wasserman and Faust 1994). If so, the number of closed triads would be higher than by chance, which also suggest that the number of motifs with a coordinating actor should also be higher. However, the ineffective motifs that describe uncoordinated responses to ecological processes are less common than expected, and the “ideal” motifs in both models (Fig. 2a, motifs c and d; Fig. 2, motifs g–l), are overrepresented, therefore reflecting a drive for public governance effectiveness in matters of conservation of shared resources. Even though we do not have data to causally link these favorable patterns of motifs with a deliberate intention to increase governance effectiveness, the issue of ecological connectivity has been given much attention in the Stockholm County (Stockholm County Council 2012) as has the need for municipalities to collaborate around these trans-boundary issues (Ingo et al. 2006). Hence, we suggest that these efforts have yielded results, and at least partly help to explain our results. It should, however, also be stated that a previous study (Bergsten et al. 2014) found no support for municipalities collaborating *just* because of ecological connections. Municipalities do, however, tend to collaborate with their neighbors and since neighbors are often connected ecologically due to spatial proximity, this propensity for resource management can also help explain the observed pattern.

Focusing on the individual positions of the municipalities, we observe some interesting patterns. For example, the position of Nykvarn and Haninge in the PCA plot, indicating heavy involvement as coordinating actors among already connected others (Fig. 6), may partially be a result of being members of the well-established Södertörn group. This group, which includes the eight southernmost municipalities, engages in joint infrastructure projects and nature conservation (Ingo et al. 2006). Furthermore, Stockholm is the central and the most prevailing coordinating actor linking otherwise unconnected councils in the study area. Stockholm comprises half of the study areas' total human population and hosts a relatively large municipal organization. In contrast to Nykvarn and Haninge, Stockholm’s social network includes municipalities both in the southern and northern part of the study area, which do not collaborate with each other. Hence, it seems plausible to assume that Stockholm plays a particularly important role as coordinator in the study area, and that without Stockholm, there would be minimal social links between the northern and the southern municipalities. Similarly, but on a smaller scale, Täby has links to both Vallentuna and Österåker, for which we did not observe a direct collaboration despite a high number of inter-municipal wetlands.

Further analysis of this motif suite in terms of entanglement (such as when a possible propensity to form closed social triads lead to an increase in the occurrences of more complex 5 motifs with coordinating actors described above) will require a more sophisticated technique. Since the more complex 5-node motifs are a composite of 2–4-node motifs, a full-fledged statistical analysis requires that the frequencies of all the dependent subcomponents be evaluated (see also Milo et al. 2002). Recent developments in social network analysis have made that possible (Multilevel Exponential Random Graph Modeling, see Wang et al. 2013). However, the capacity to include the 5-node motifs shown in Fig. 2 is not possible at present nor is the possibility to single out the actors’ individual positions in the different motifs (Table 4). Hence, while we can describe the general trends observed across the conceptual model, we are unable to quantify the impact of “simpler” subcomponent to the frequency of occurrence of the more complex 5-node motifs. Until further progress has been made to statistically disentangle 5-node motifs from simpler motifs, we specifically argue that significant caution is needed when interpreting the frequencies of the different 5-node coordinating actors motifs.

While the analysis of regional patterns of governance is instructive, we do not imply here that a council must adopt a particular motif configuration in order to be effective. Rather each council needs to assess if the governance structures that apply to ecological systems match the ecological processes in ways that ensure adequate management (Boyd et al. 2015). Given the limited resources and in-house expertise, the involvement of a coordinating actor such as neighboring council or specially established government agency may be a preferred operational structure despite the additional coordination burden.

Importantly, the social network was constructed through a questionnaire study that essentially focused on the engagement of stakeholders in dialog on wetlands and not on the number of joint activities undertaken by the cooperating councils (Bergsten et al. 2014). Clearly, the assumption that the existence of social links positively influences activities that are in the best interest of the environment is assumed here. Detailed monitoring of the activities and the wetland ‘health’ is necessary to validate this assumption and also that the committed actions are ecologically appropriate (Guerrero et al. 2013). While we admit that the approach applied here describing the social and ecological linkages is dependent on several assumptions, the primary goal of this research is to stimulate further investigations of effective governance structures in a detailed, explicit, and theoretically informed manner.

## Electronic supplementary material

Below is the link to the electronic supplementary material.

Supplementary material 2 (PDF 634 kb)
