# Supplementary material for: Closing the collaborative gap: Aligning social and ecological connectivity for better management of interconnected wetlands
Source: Ambio. 2015 Jan 9;44(Suppl 1):138–48. doi: 10.1007/s13280-014-0605-9 (PMC4289001; doi:10.1007/s13280-014-0605-9)
Supplement: Supplementary file 1 — Supplementary material 2 (PDF 634 kb) [file 13280_2014_605_MOESM1_ESM.pdf]

AMBIO

Electronic Supplementary Material

Closing the collaborative gap: Aligning social and ecological connectivity for better management of interconnected wetlands

Stuart Kininmonth, Arvid Bergsten, Örjan Bodin

1. Case Study

1.1. Social network - inter-municipal collaboration

To explain what we meant with "inter-municipal collaboration" we included examples of different types of wetland-related collaborative activities, viz., information exchange; co-production of ecological knowledge like reports and field assessments; policy work and joint planning and management. Our analysis assumes that the presence of such a collaboration means a greater chance for sustainable use and protection of a ecological resource that is shared, or two resources that are ecologically linked, compared to when there is no communication between actors. The mechanisms in force might be for example increased awareness, collaborative reflection or practical adaption *vis-à-vis* ecological processes. Instances of such joint matching activities in the data include strategy development, conservation planning, water management, collection and sharing of field data; communication of planned developments or conservation actions, and habitat restoration.

In the social network we limit the interactions to reciprocal links, which mean that collaborations mentioned by only one of the two involved municipalities are excluded in the analysis. This approach enhances the fidelity of the collaboration network by reducing the risk of including weak collaborative ties and limits unintended effects of respondents' potential misinterpretations of the survey questions.

1.2. Ecological network – Organism based justification of connectivity

Our model represents two scales of connectivity; local level movement confined essentially to the area where the organisms frequently move around to forage and to adjust to seasonal variations, and rare dispersal events that bring together local populations into metapopulations (Bergsten et al. 2014; Bergsten 2014). The former scale is captured by our representation of an ecological node as a zone for regular amphibian movement, i.e., a continuous area in which an organism is assumed to be able to reach and utilize a minimum habitat area (an area that equals or exceeds the minimal requirements for a suitable home range)

(cf. Ray et al. 2002). We evaluate 3, 6 or 12 hectares as a minimum wetland area requirement for such zones to be perceived hospitable (table 2 and figure 3). These movement zones consist of one or more wetlands that may be separated up to 2 km (between large wetlands) and where the total area of these wetlands sum up to at least 3, 6 or 12 hectares. Genetic studies on frog population structure have found that the effective sizes of frog populations often encompass several breeding ponds up to 2 km apart, e.g., for the Common toad *Bufo bufo* (Scribner et al. 2001; Ray et al. 2002; Janin et al. 2009). Angelone et al. (2011) found that European tree frogs *Hyla arborea* were able to cover distances up to 2 km on a regular basis without reduced gene flow between local populations. Dispersal studies has demonstrated that many frog species are capable of moving regularly up to 2 km, including the Common toad (Sinsch 1988), the European tree frog (Vos et al. 2000; Angelone et al. 2011), the European firebelly toad *Bombina bombina* (Edenhamm et al. 1999) and the Common frog *Rana temporaria* (Kovar et al. 2009). Furthermore, a review on 90 species suggested that frogs display average movements up 2.0 km (Smith and Green 2005).

Genetic differentiation between frog populations increases at longer distances between habitat patches, corresponding to the tail of the dispersal curve where dispersal events are rare (Manier and Arnold 2006; Knopp and Merilä 2009). Our analysis evaluates 4, 8 and 13 km as hypothetical threshold distances for those rare dispersal events that determine metapopulation connectivity on a scale larger than the movement zones (the ecological nodes in our model). These distances apply to the European tree frog for example (Vos et al. 2000; Angelone et al. 2011). Smith and Green (2005) suggested that frog populations isolated by distances approaching 11-13 km are more likely to exhibit metapopulation structure than less isolated populations.

To identify potential movement zones (ecological nodes), we adopted the method outlined by Bergsten et al. (2014) and created a raster map with 25-meter resolution by calculating the mean wetland density for each raster cell in a circular unweighted window with 2 km diameter (i.e. our estimated short-term maximum movement distance). This moving-window algorithm was repeated to generate a smoother density map that more accurately represented the actual distribution of the wetlands in the study area.

2. Networks representing the 3 dispersal intervals and 3 home range sizes

The home ranges of 3, 6 and 12 hectares were combined with dispersal threshold distances of 4, 8 and 13 kilometers to create 9 networks. The basic metrics of these networks are shown in Table S1. Each network is also plotted in figure S1 using the 'Fruchterman Reingold' algorithm (Fruchterman and Reingold 1991) for layout configuration. The area corresponds to all municipalities in the Stockholm County except Norrtälje which we excluded because previous research has indicated that >99% of the ecological connectivity (measured as habitat availability) of Norrtälje's extensive wetland systems was attributed to ecological processes within the municipal boundary (Bergsten 2014). Hence wetland management in Norrtälje is principally an internal governance question.

Table S1 The metrics for the 9 networks used to model the wetland governance socio-ecological system. For all networks there were 25 Council nodes in addition to the wetland nodes.

Home range	Dispersal distance	Average degree	Edge number	Wetland nodes	Edge density	Component number
3ha	4km	3.74	200	82	0.04	1
3ha	8km	5.87	314	82	0.06	1
3ha	13km	9.33	499	82	0.09	1
6ha	4km	4.00	274	112	0.03	2
6ha	8km	6.76	463	112	0.05	1
6ha	13km	11.68	800	112	0.09	1
12ha	4km	4.00	282	116	0.03	3
12ha	8km	7.11	501	116	0.05	2
12ha	13km	12.45	878	116	0.09	1

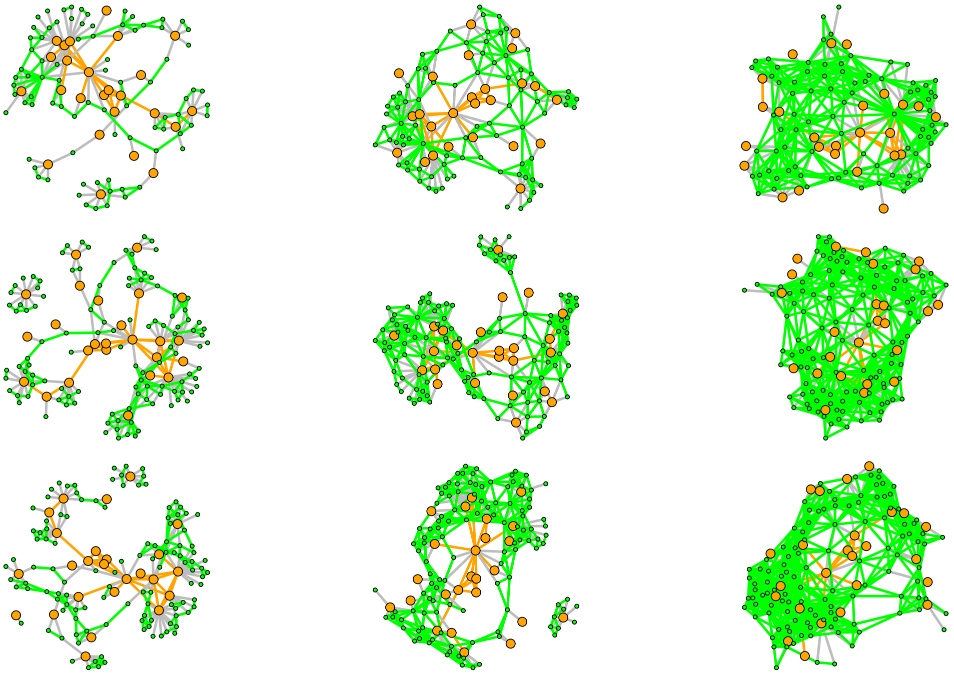

Fig. S1 Network plots representing the wetland governance case for home ranges of 3 ha (top row), 6 ha (middle row) and 12 ha (bottom row) and dispersal distances of 4km (left column), 8km (center column) and 13km (right column). Larger orange circles represent Municipal Councils while green circles represent shared wetlands. Orange links show Council to Council social interactions, grey links show resource governing connections while green links show wetland to wetland ecological connections

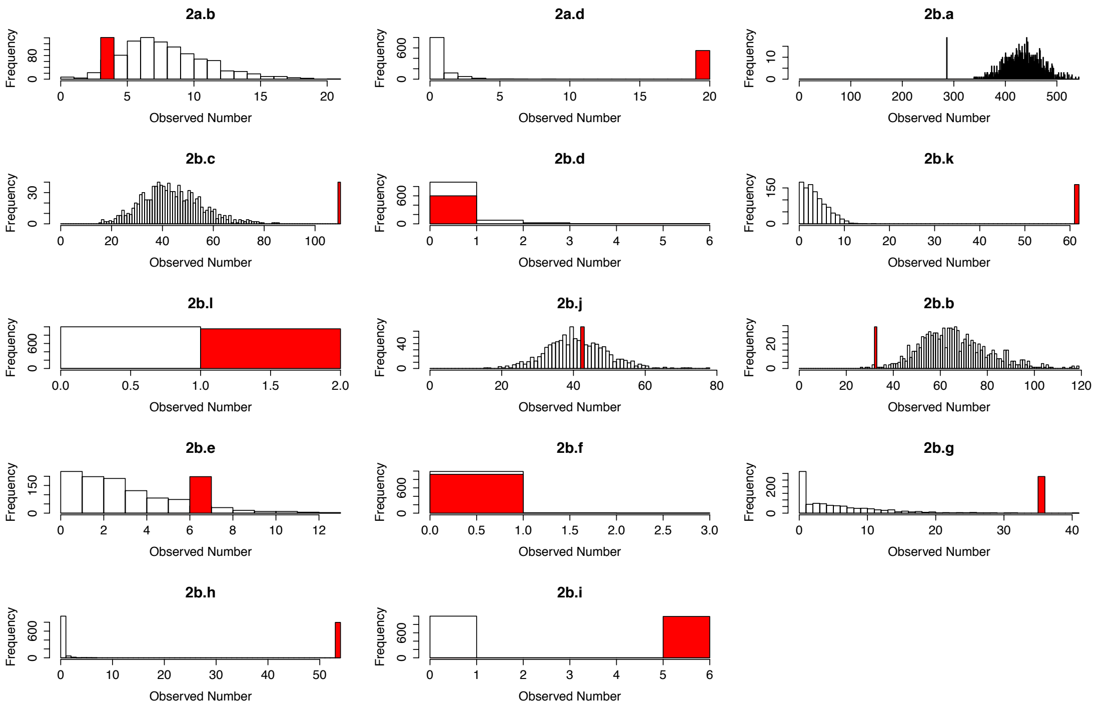

Fig. S2 Histograms for each motif in figure 2 for the 1000 random simulations. Motifs 2a.a and 2a.c are shown in figure 5. The observed number for the simulation of 3 ha home range and 13km dispersal threshold is shown by the red bar.

3. References

Angelone, S., F. Kienast, and R. Holderegger. 2011. Where movement happens: scale-dependent landscape effects on genetic differentiation in the European tree frog. *Ecography* 34: 714–722. doi:10.1111/j.1600-0587.2010.06494.x.

Bergsten, A. 2014. Spatial complexity and fit between ecology and management, Doctoral Thesis. Stockholm University.

Bergsten, A., D. Galafassi, and Ö. Bodin. 2014. The problem of fit in socio-ecological systems: Detecting spatial mismatches between ecological connectivity and land management in an urban region. *Ecology and Society* in press: 0.

Edenhamm, P., M. L. Ekendahl, and P. Pamilo. 1999. *Spridningsförmåga hos svenska växter och djur*. *Naturvårdsverket*. Stockholm: Naturvårdsverket.

Fruchterman, T. M. J., and E. M. Reingold. 1991. Graph Drawing by Force-directed Placement 21: 1129–1164.

Janin, A., J.-P. Léna, N. Ray, C. Delacourt, P. Allemand, and P. Joly. 2009. Assessing landscape connectivity with calibrated cost-distance modelling: predicting common toad distribution in a context of spreading agriculture. *Journal of Applied Ecology* 46: 833–841. doi:10.1111/j.1365-2664.2009.01665.x.

Knopp, T., and J. Merilä. 2009. Microsatellite variation and population structure of the moor frog (Rana arvalis) in Scandinavia. *Molecular ecology* 18: 2996–3005. doi:10.1111/j.1365-294X.2009.04252.x.

Kovar, R., M. Brabec, R. Vita, and R. Bocek. 2009. Spring migration distances of some Central European amphibian species. *Amphibia-Reptilia* 30: 367–378. doi:10.1163/156853809788795236.

Manier, M. K., and S. J. Arnold. 2006. Ecological correlates of population genetic structure: a comparative approach using a vertebrate metacommunity. *Proceedings. Biological sciences / The Royal Society* 273: 3001–9. doi:10.1098/rspb.2006.3678.

Ray, N., A. Lehmann, and P. Joly. 2002. Modeling spatial distribution of amphibian populations: a GIS approach based on habitat matrix permeability. *Biodiversity & Conservation* 11: 2143–2165.

Scribner, K. T., J. . Arntzen, N. Cruddace, R. . Oldham, and T. Burke. 2001. Environmental correlates of toad abundance and population genetic diversity. *Biological Conservation* 98: 201–210. doi:10.1016/S0006-3207(00)00155-5.

Sinsch, U. 1988. Seasonal changes in the migratory behaviour of the toad Bufo bufo : direction and magnitude of movements. *Oecologia* 76: 390–398.

Smith, M. A., and D. Green. 2005. Dispersal and the metapopulation paradigm in amphibian ecology and conservation: are all amphibian populations metapopulations? *Ecography* 28: 110–128.

Vos, C., C. Braak, and W. Nieuwenhuizen. 2000. Incidence Function Modelling and Conservation of the Tree Frog Hyla arborea in the Netherlands. *Ecological Bulletins* 48: 165–180.
